# Endoscopic rhizotomy for chronic lumbar zygapophysial joint pain

**DOI:** 10.1186/s13018-019-1533-y

**Published:** 2020-01-03

**Authors:** Yuntao Xue, Tao Ding, Dajie Wang, Jianli Zhao, Huilin Yang, Xiaofeng Gu, Dehong Feng, Yafeng Zhang, Hao Liu, Fenglin Tang, Wanyi Wang, Miao Lu, Chao Wu

**Affiliations:** 1 Department of Orthopaedics, The Affiliated Wuxi People’s Hospital of Nanjing Medical University, No.299 Qingyang Road, Wuxi City, Jiangsu Province China; 2 Department of Anesthesiology, Thomas Jefferson University Hospital, 834 Chestnut St. T150, Philadelphia, PA 19107 USA; 3 Department of Anesthesiology, First Hospital of Shanxi Medical University, Taiyuan, Shanxi China; 4 Department of Orthopedic Surgery, The First Affiliated Hospital of Soochow University, Suzhou, Jiangsu China; 5 Department of Orthopaedics, Wuxi Affiliated Hospital of Nanjing University of Chinese Medicine, Wuxi, Jiangsu China; 6 Department of Orthopaedics, Wuxi Hand Surgery Hospital, Wuxi, Jiangsu China; 7 ECG Room, Wuxi 3rd People’s Hospital, Wuxi, Jiangsu China

**Keywords:** Endoscope, Lumbar medial branch nerves, Medial branches of the dorsal rami, Lumbar facet syndrome, Chronic low back pain

## Abstract

**Background:**

Chronic lumbar zygapophysial joint pain is a common cause of chronic low back pain. Percutaneous radiofrequency ablation (RFA) is one of the effective management options; however, the results from the traditional RFA need to be improved in certain cases. The aim of this study is to investigate the effect of percutaneous radiofrequency ablation under endoscopic guidance (ERFA) for chronic low back pain secondary to facet joint arthritis.

**Methods:**

This is a prospective study enrolled 60 patients. The cases were randomized into two groups: 30 patients in the control group underwent traditional percutaneous radiofrequency ablation, others underwent ERFA. The lumbar visual analog scale (VAS), MacNab score, and postoperative complications were used to evaluate the outcomes. All outcome assessments were performed at postoperative 1 day, 1 month, 3 months, 6 months, and 12 months.

**Results:**

There was no difference between the two groups in preoperative VAS (*P* > 0.05). VAS scores, except the postoperative first day, in all other postoperative time points were significantly lower than preoperative values each in both groups (*P* < 0.05). There was no significant difference between the two groups in VAS at 1 day, 1 month, and 3 months after surgery (*P* > 0.05). However, the EFRA demonstrated significant benefits at the time points of 3 months and 6 months (*P* > 0.05). The MacNab scores of 1-year follow-up in the ERFA group were higher than that in the control group (*P* < 0.05). The incidence of complications in the ERFA group was significantly less than that in the control group (*P* < 0.05).

**Conclusions:**

ERFA may achieve more accurate and definite denervation on the nerves, which leads to longer lasting pain relief.

## Background

The lumbar zygapophysial joint is a synovial joint between two adjacent vertebrae. The joint capsule and surrounding tissues are densely covered with nerve terminal which mainly originate from the lumbar dorsal medial branch [1].

Chronic lumbar zygapophysial joint pain refers to low back pain with a course longer than 3 months accompanied by radiating pain down to the buttocks and legs [2]. Medial branch block (MBB) is a reliable approach to confirm facet joints as the source of back pain [3].

Percutaneous radiofrequency nerve ablation is an effective management with the problem of neural anatomic variation and regeneration [4]. This study was designed to evaluate the efficacy and safety of radiofrequency ablation under endoscopic guidance (ERFA) in the treatment of chronic lumbar zygapophysial joint pain.

## Methods

**Fig. 1:**
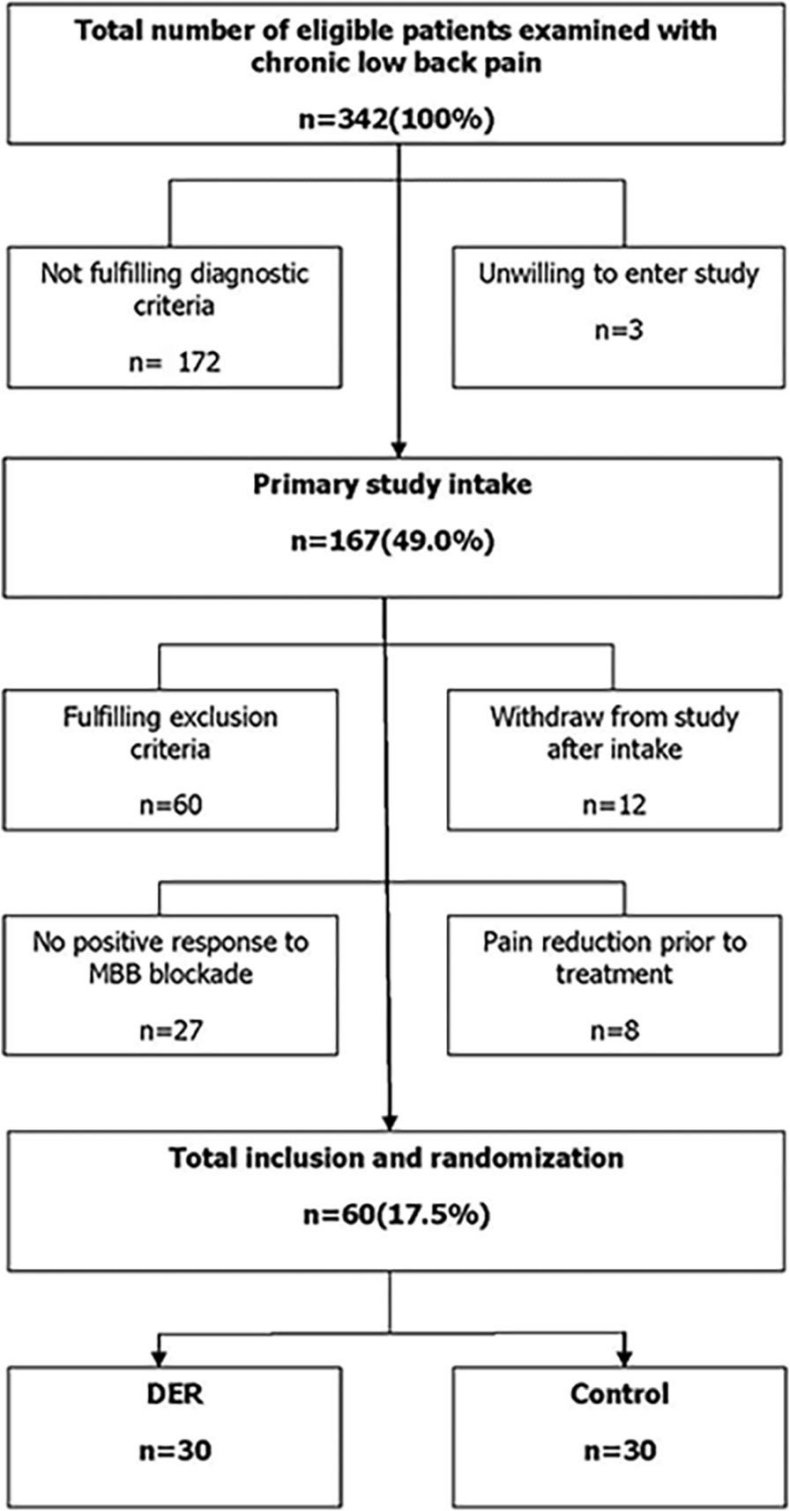
Inclusion and exclusion process of eligible patients examined with chronic low back pain

Inclusion criteria are as follows: (1) chronic low back pain with a course longer than 3 months; (2) failed 2 months of conservative treatment including physical therapy and NSAIDs; (3) no change of lower limb sensation, movement and nervous reflex, no disorder of defecation or urination; (4) imaging feature: hyperplasia of the zygapophysial joints, osteophytes of the articular process, narrowing of the joint space (less than 2~4 mm), osteoarthritis, asymmetry, and intra-articular vacuum phenomenon; (5) more than 80% pain relief after controlled differential MBB with 0.5 ml 2% lidocaine and 1% bupivacaine at transitional part of superior articular process and transverse process in a week, respectively. The use of long-acting local anesthetic drugs to obtain longer pain relief time than short-acting local anesthetic drugs means true-positive response (Fig. 1).

Exclusion criteria are as follows: (1) tuberculosis, infections, tumors, and fractures, etc.; (2) spinal canal compromise, lumbar disc herniation, and spinal stenosis; (3) hematological diseases, diabetes, and gastrointestinal ulcers; and (4) age < 18 years or pregnant women.

Participants: A total of 60 patients with chronic lumbar zygapophysial joint pain who fulfilled inclusion criteria were randomized into the ERFA group and control group according to the principle of randomized control and informed consent. Radiofrequency ablation was performed on 30 patients in the control group while endoscopic radiofrequency rhizotomy was performed for the 30 patients in the ERFA group. The demographic characteristics such as age, gender, body mass index, history of lumbar surgery, and low back pain in both groups are shown in Table 1. There was no significant difference between the two groups in gender, level of zygapophysial joint pain source, and history of lumbar surgery (chi-square test, *P* > 0.05). There was no significant difference in age, duration of back pain, and body mass index (*t* test, *P* > 0.05).

**Table 1:** Demographic characteristics

Index	ERFA group	Control group	*P* value
Cases (*n*)	30	30	
Gender (male/female)	16/14	17/13	0.795
Age (years)	65.73 ± 7.62	64.78 ± 6.62	0.607
Duration of back pain (months)	46.83 ± 11.43	46.71 ± 11.21	0.967
History of lumbar surgery (yes/no)	10/20	9/21	0.781
BMI (kg/m^2^)	21.52 ± 1.32	21.44 ± 1.33	0.816
L3-4, L4-5, L5-S1	6, 16, 8	5, 17, 8	0.941

This prospective randomized trial was performed and approved by medical ethical committees, written informed consent was obtained from all patients.

### ERFA surgical methods

**Fig. 2:**
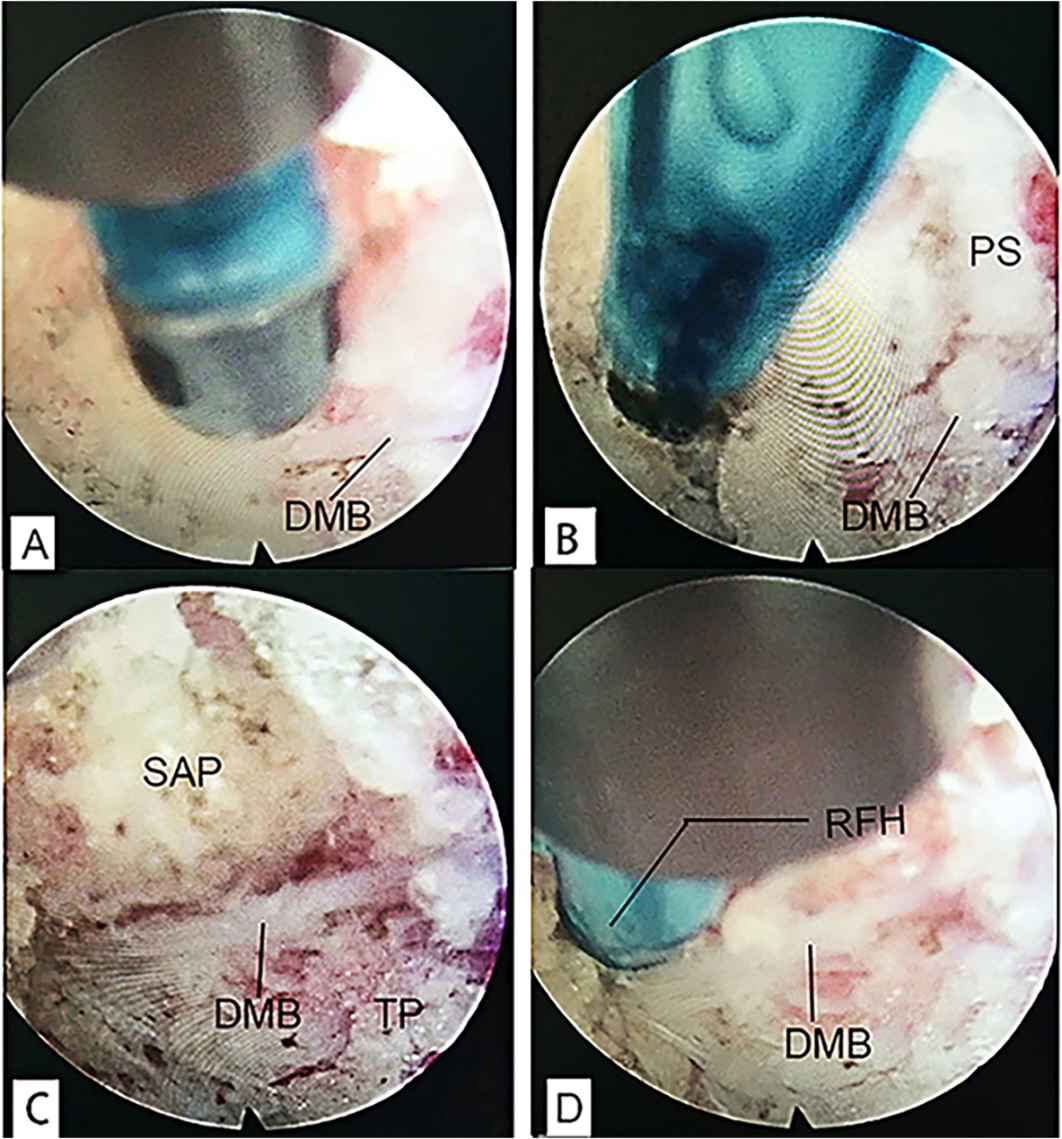
**a** Dorsal medial branch was exposed. **b** Dorsal medial branch embodied in the thick periosteum. **c** Dorsal medial branch is in the transitional position between transverse process base and superior articular process. **d** Test branch with radio frequency head and repeat pain complaint. DMB, dorsal medial branch; PS, periosteum; SAP, superior articular process; TP, transverse process; RFH, radiofrequency head

**Fig. 3:**
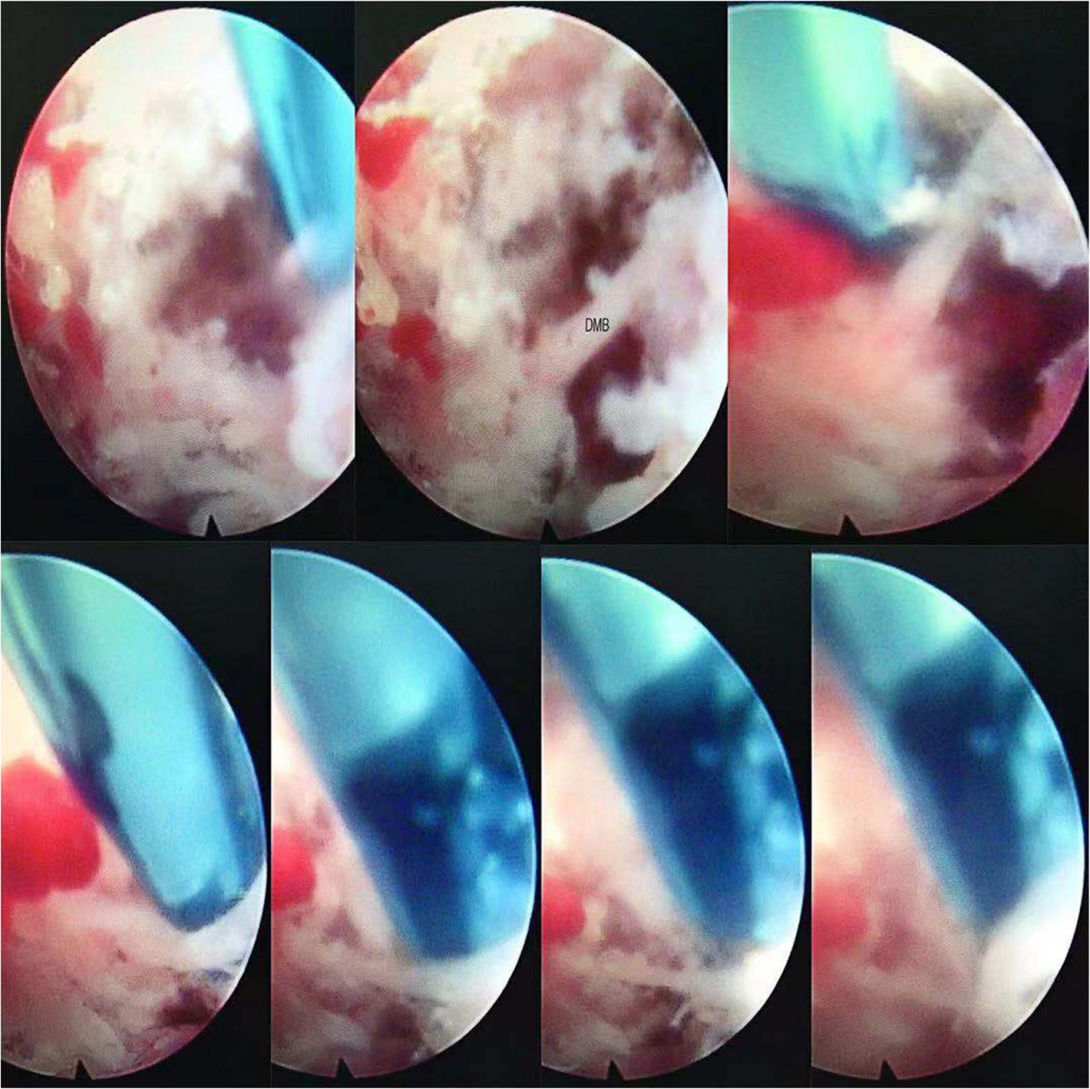
The consecutive process of dissecting and ablation of dorsal medial branch. DMB, dorsal medial branch

Operation was performed under local anesthesia. Patients were placed in the prone position with a soft pillow under the abdomen to maintain lumbar kyphosis and the abdomen unstressed. After confirming the segment (medial branch nerve of L3 and L4 are at the point of intersection of superior articular process and transverse process of L4 and L5, respectively; dorsal ramus of L5 is at the point of intersection of superior articular process and alae sacralis), C-arm was adjusted until the clear transition site of the facet joints and transverse processes are observed. Following local anesthetic injection of the puncture site on the skin, the tip of the radiofrequency needle reached the base of the transverse process, where the rigid bony was encountered. Adjust needle tip cranially sliding over the bony tissue and parallel to the medial branch. After the probe is in place, the guide needles were inserted through the radiofrequency needle, following stepwise dilation and placement of working channel. Under the endoscope, dorsal medial branch was dissected and ablated with a radio frequency cutting head. The radiofrequency denervation is performed on the severed nerve end using high frequency to avoid nerve regeneration. The soft tissue was fully ablated to expose the bone surface of dorsal transverse process and the superior articular process. A skin incision was sutured by absorbable suture material after the endoscope and working cannula were removed. Other targets were operated referring to this method. On the second postoperative day, normal life and work can be resumed (Figs. 2 and 3).

### Radiofrequency treatment

The radiofrequency ablation of medial branches of the dorsal rami was performed under local anesthesia. The patient was placed in a prone position; responsible segment and the upper segment of the lumbar spinal nerve medial branch were selected. A 22-G, 10-cm-long radiofrequency needle with the 0.5-cm bare end was punctured into the transitional part of the facet joint and transverse process under C-arm fluoroscopy. After location, sensory testing and motor testing were performed. The sensory test was performed with frequency at 50 Hz. The sensory test was successful if the chief complaint pain area had slight pain, warmth, bulging sensation, or string numbness, without severe pain of lower extremities. If motor test with low-frequency stimulation at 2 Hz did not induce muscle twitching in lower extremities, the tip position was considered accurate and ablation was feasible. The ablation parameters were set to 80 °C, lasting 60 s once, and 90 °C, 80 s one more time.

### Efficacy assessment

The lumbar visual analog scale (VAS) scores were recorded previous to treatment, posterior to second MBB, 1 day, and 1, 3, 6, and 12 months posterior to treatment. MacNab scores at the 12 months after treatment were recorded.

### Statistical analysis

All analyses were performed using SPSS 21.0 (SPSS, Chicago, IL, USA). One-way ANOVA analysis was used to compare preoperative and postoperative VAS scores. *T* test was used to compare the VAS scores at each time point after surgery. The comparison of MacNab score and follow-up complications among groups was done with chi-square test. Differences were considered statistically significant when *P* < 0.05.

## Results

The average duration of ERFA and radiofrequency ablation was 55 min (35–75 min) and 33 min (15–51 min), respectively. The duration of ERFA is longer than that of radiofrequency ablation (*P* < 0.05). Cases underwent ERFA returned to daily life after 3–7-day wound care and observation. There were multiple anatomic variations of medial dorsal rami observed. There was no significant difference in pre MBB VAS scores (*P* > 0.05). Because of the pain in the wound, the VAS score of back pain at the first day after surgery was higher than that before surgery in both two groups (*P* < 0.05). Post MBB and postoperative VAS scores were significantly lower than pre MBB ones at 1 month, 3 months,6 months, and 12 months (*P* < 0.05); there was no significant difference in VAS scores between the ERFA group and the control group at 1 day, 1 month, and 3 months (*P* > 0.05). In the ERFA group, the low back pain showed a lower score at postoperative 6 and 12 months (*P* < 0.05). There was no significant difference in VAS scores at all time points after surgery except postoperative 1 day in the ERFA group (*P* > 0.05), VAS scores of the control group decreased firstly and then increased with time after radiofrequency treatment (Table 2). One-year post-operation MacNab score of ERFA was excellent in 21 cases, good in 8 cases, and fair in 1 case, with the excellent and good rate at 96.7%. The score of the control group was excellent in 13 cases, good in 8 cases, fair in 5 cases, and poor in 4 cases, with the excellent and good rate at 70%. The excellent and good rate of the ERFA group was significantly higher than that of the control group (*P* < 0.05) (Table 3). The incidence rate of operative complications in ERFA group was lower than that in the control group (*P* < 0.05) (Table 4).

**Table 2:** VAS score at different time points (x ± s)

Time point	ERFA group	Control group	*t* value	*P* value
Cases (*n*)	30	30		
Pre MBB	7.70 ± 0.85	7.56 ± 0.61	0.733	0.469
Post MBB	0.49 ± 0.48	0.50 ± 0.53	0.766	0.940
Postoperative 1 day	8.69 ± 1.07	8.70 ± 0.75	0.419	0.967
Postoperative 1 month	3.84 ± 0.83	3.83 ± 0.82	0.469	0.963
Postoperative 3 months	3.59 ± 0.93	3.55 ± 1.11	0.151	0.880
Postoperative 6 months	3.61 ± 1.02	5.75 ± 1.67	5.990	< 0.001
Postoperative 12 months	3.69 ± 1.13	5.36 ± 1.38	5.129	< 0.001
*F* value	273.170	200.003		
*P* value	<0.001	<0.001

**Table 3:** MacNab scores at the 12 months after surgery (*n*)

MacNab scores	ERFA group	Control group	*χ*^2^	*P* value
Cases (*n*)	30	30		
Excellent	21	13		
Good	8	8		
Fair	1	5		
Poor	0	4		
The excellent and good rate	96.70%	70%	7.680	0.006

**Table 4:** Comparison of follow-up complications between two groups (*n*)

Complications	ERFA group	Control group	*χ*^2^	*P* value
Cases	30	30		
Lack of skin sensation	1	3		
Analgesia	1	6		
Total	2	9	5.455	0.020

## Discussion

Chronic low back pain is one of the common diseases in orthopedic outpatient clinics, and low back pain secondary to facet joint arthritis accounts for about 30% (circa. 15 to 45%) of patients with chronic low back pain [5]. The lumbar joint synovial membrane and joint capsule distribution have abundant nerve endings, which sense and transmit pain information. These endings mainly originate from the medial branch of the dorsal branch of the spinal nerve. After the posterior branch of the spinal nerve exit from the intervertebral foramen, it divides into medial and lateral branches. The posterior medial branch runs on the upper edge of the transverse process of the lower vertebral body and the outside of the upper articular process. After it continues its routine against the upper articular process, it enters a fibro osseous canal located between the mammillary and the accessory processes, giving off fibers to facet joints and surrounding muscles [6]. When the spine degenerates, the increased stress in the facet joints will impinge the synovial membrane folds, stimulate the sensory receptors in the joint capsule, and cause lumbar zygapophysial joint pain through the posterior medial branch of the spinal nerves. In clinical practice, it usually presents with radiating pain, unclear localized physical sign of low back pain, without symptoms of nerve root compression.

Controlled MBB is the only mean diagnostic method for the diagnosis of lumbar zygapophysial joint pain. The false-positive rate of single uncontrolled MBB is between 22 and 32% [7]. Controlled MBB is used to reduce the incidence of false-positives, which inject different local anesthetic drugs (lidocaine and bupivacaine) at different times. A true-positive response means the use of long-acting local anesthetic drugs to obtain longer pain relief time than short-acting local anesthetic drugs, the validity of lidocaine is generally more than 1 h, and the efficacy of bupivacaine is more than 2 h. The efficiency of conservative treatment is unsatisfactory and temporary for a specific part of patients. Repeated medication administration increases the side effect risk of elder patients with organic diseases [8]. Nerve block therapy of low back pain plays a symptomatic anti-inflammatory effect and shows a short-term good outcome [9]. However, its efficacy does not reach the clinical therapeutic goal, and the long-term efficacy is poor.

Percutaneous radiofrequency ablation of spinal nerves is effective; however, the long-term efficacy is unsatisfactory due to the variation of dorsal medial branch and the recurrence caused by nerve regeneration [10].

Indications for EFRA include (1) chronic lumbar zygapophysial joint pain with a definite diagnosis of controlled MBB and patient has a pronounced surgical aspiration and (2) failure or short-time recurrence after 6-week standard conservative treatment and percutaneous radiofrequency ablation. In the current study, the therapeutic effect of ERFA is superior to percutaneous radiofrequency ablation. The reasons may include direct vision of the variability of the nerve under endoscopy [11], with direct vision under endoscopy, ablation can be more accurate and reduce nerve root injury rate, reducing the incidence of sensory loss or analgesia of skin; nerve regeneration is fairly rare after a long segment ablation and additional radiofrequency denervation on the severed nerve end, the long-term efficacy is better [12]. The sensation and motor tests with radiofrequency needle contribute to determine the accurate position that endoscopic channel placement was immediate and prompt.

Compared with radiofrequency ablation, ERFA also has corresponding disadvantages, such as longer duration of procedure, lengthened recovery time, and increased cost. Besides, insertion of the working channel may cause potential cosmetic scarring. But given the advantages of the procedure, these disadvantages are also acceptable.

## Conclusions

In summary, radiofrequency needle guiding ERFA has advantages of more accurate positioning, more thorough denervation, fewer complications, lower risk, and better long-term efficacy up to 5 years post-procedure. The surgery may be used in selected patients. However, this study involves a small sample size and short follow-up time. The long-term efficacy needs further follow-up studies.

## Data Availability

Upon request, raw data can be provided.

## Abbreviations

ERFA: Radiofrequency ablation under endoscopic guidance
MBB: Medial branch block
RFA: Radiofrequency ablation
VAS: The lumbar visual analog scale
